# Transient peak of p53 induction persists at low radiation doses in MCF-7 cells

**DOI:** 10.1007/s11033-026-12787-7

**Published:** 2026-10-01

**Authors:** Irina Danilova, Jana Klementová, Šárka Jarošová, Pavel Kundrát, Martina Zíková, Marie Davídková

**Affiliations:** 1 Faculty of Nuclear Sciences and Physical Engineering, Czech Technical University in Prague, Břehová 78/7, Praha 1, 110 00 Czech Republic; 2 Department of Radiation Dosimetry, Nuclear Physics Institute of the Czech Academy of Sciences, Na Truhlářce 39/64, Praha 8, 180 00 Czech Republic; 3 Institute of Molecular Genetics of the Czech Academy of Sciences, Vídeňská 1083, 142 20 Praha 4, Czech Republic; 4 Faculty of Science, Charles University, Albertov 6, Praha 2, 128 00 Czech Republic

**Keywords:** p53 protein, Tumour suppressor, MDM2 oscillation, Transient peak expression, Breast cancer cells MCF-7, Ionizing radiation

## Abstract

**Background:**

The p53 protein is a central tumour suppressor regulating numerous genes involved in cell cycle control, DNA repair, and apoptosis. Mutations in p53 occur in over half of all cancers, disrupting these regulatory pathways and enhancing tumour progression. Many cell lines, including MCF-7 breast cancer cells, exhibit oscillatory p53 and MDM2 activity, a phenomenon linked to radiosensitivity and the cellular response to ionizing radiation.

**Methods and Results:**

We investigated the dose-dependent dynamics of p53 activation in MCF-7 cells exposed to γ-radiation in the dose range of 0.1 to 8 Gy. Using western blot analysis and real-time quantitative PCR, we examined dynamics of the expression of p53 and its downstream targets, including *CDKN1A*, *GADD45A*, and *MDM2* genes. Our findings reveal that while oscillations in p53 and its downstream targets are prominent at high radiation doses, transient peak expression persists even at doses as low as 0.1 Gy, though with delayed onset and diminished amplitude.

**Conclusions:**

The results extend the knowledge on p53 dynamics down to 0.1 Gy. They challenge the previous models that predicted the absence of oscillations at low radiation doses.

**Supplementary Information:**

The online version contains supplementary material available at https://doi.org/10.1007/s11033-026-12787-7.

## Introduction

The tumour suppressor protein p53 mediates the cellular response to various stress stimuli including ionizing radiation. Regulatory functions of p53 are essential for preserving genomic integrity and preventing neoplastic transformation [1]. The significance of the p53 protein in carcinogenesis is underscored by the fact that in about half of all tumours, p53 is directly inactivated due to mutations in the p53 gene. In many other cases, its inactivation is caused indirectly, often through other proteins [2]. The primary function of the transcription factor p53 is to regulate the expression of several hundred genes that control cell cycle checkpoints, cell cycle arrest, DNA repair or cellular senescence, and initiate programmed cell death, known as apoptosis [3, 4]. Under normal conditions, p53 is present in cells at low concentrations in an inactive or minimally active state [5]. When DNA damage occurs, the level of active p53 protein increases, which via binding to specific DNA sequences eventually leads to G1 cell cycle arrest, allowing time for DNA repair. Therefore, p53 activation following irradiation can result in the cessation of cell proliferation or in direct cell death [6].

**Fig. 1:**
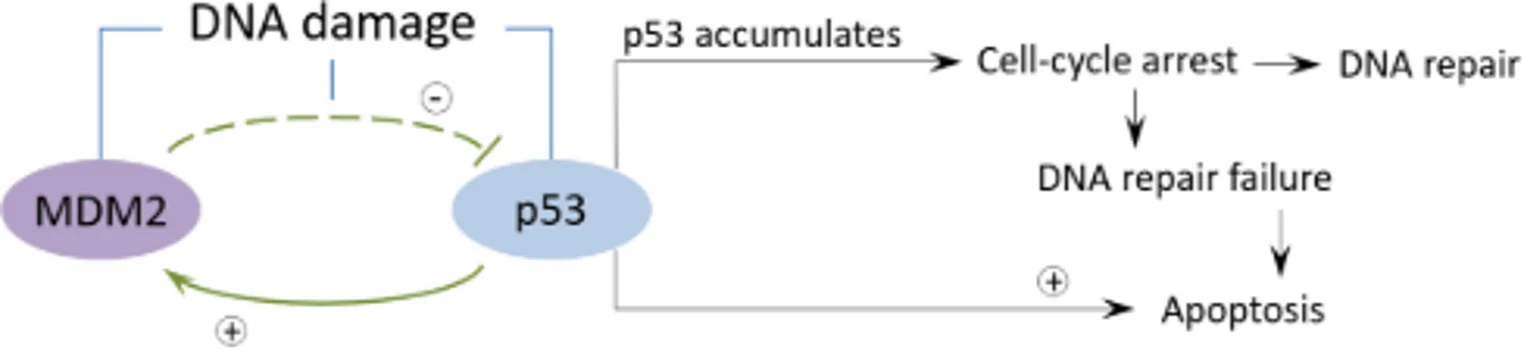
Scheme of the p53-MDM2 negative feedback loop

Within cells, levels of p53 protein are tightly controlled by several regulatory feedback loops that direct its stability and degradation. A key regulator of p53 is the E3 ubiquitin ligase mouse double minute 2 (MDM2). MDM2 exhibits a dual relationship with p53. On the one hand, p53 positively regulates MDM2 by activating MDM2 transcription; on the other hand, MDM2 negatively regulates p53 by promoting its ubiquitination and proteolytic degradation. This defines a negative feedback loop (Fig. 1), which keeps p53 levels low under unstressed conditions [7–9]. However, in response to DNA damage, PI3-like kinases ataxia telangiectasia mutated (ATM) or ataxia telangiectasia and Rad3-related (ATR) disrupt the p53-MDM2 interaction by phosphorylation of MDM2 and p53. This leads to p53 stabilization and accumulation in the nucleus where p53 induces the expression of target genes [10].

The p53-MDM2 negative feedback loop cooperates with other aspects of p53 regulation, resulting in pulsatile dynamics following DNA damage [11–13]. It was shown by western blot analysis that the p53 response to DNA double-strand breaks (DSBs) caused by γ-radiation in MCF-7 breast carcinoma cells showed a damped oscillatory response, with sequential peaks of p53 levels decreasing over time following the damage [11]. Subsequent single-cell analysis showed that within individual cells, p53 oscillations were almost undamped, with the concentration of p53 reaching similar levels for each oscillatory pulse over a few days [12]. Interestingly, the average amplitude, duration, and frequency of p53 pulses remained constant regardless of the irradiation dose, while the fraction of cells exhibiting oscillations as well as the number of pulses, and thus the overall duration of oscillations, increased with radiation dose [12, 13].

In further experiments, p53 oscillation induced by X-ray, 𝛾-radiation, or the radiomimetic drug neocarzinostatin was observed in vivo in mice [14], in vitro in various human cell lines [15, 16], and across cell lines from different species [17]. Analysis of p53 dynamics in response to X-ray-induced DNA damage in twelve well-established human cancer cell lines showed that the dynamics ranged from relatively consistent oscillations to a single large pulse [16]. Using a combined modelling and experimental approach, it was shown that these different dynamics mainly depend on the efficiency of the DNA repair process and the strength of the activity of the damage-sensing kinase ATM [16]. Additionally, a polymorphism in the MDM2 promoter in human cancer cell lines may also disrupt the regulation of the p53 protein and the observed oscillation [15]. Comparing p53 oscillations across cells of different species suggests that while the structural dynamics of p53 are conserved, the oscillation period may vary. The period of p53 oscillations was comparable in human, monkey, and dog cells, but in mouse cells, p53 oscillates more rapidly due to faster degradation of p53 and stronger transcription of MDM2 [17].

Since p53 regulates plenty of genes involved in cell fate decisions, its oscillations may provide a mechanism to generate distinct temporal responses in target genes, including pulsing and rising dynamics, depending on their mRNA decay rates. Short mRNA half-lives result in pulsatile gene expression, while long-lived mRNAs allow continuous transcript accumulation throughout the DNA damage response [18]. The modulation of p53 pulse amplitude, duration, and frequency differentially regulates the p53-responsive gene promoters such as *MDM2* and cyclin kinase inhibitor 1 (*CDKN1A*) which play a critical role in determining cell fate decisions [19].

The dynamics of the p53 protein in response to low-dose ionizing radiation (< 1 Gy) have not yet been precisely described. It provides mechanistic insights with potential impact on radiation protection and optimisation of radiotherapy strategies. Mathematical modelling suggests that when a cellular stress signal does not exceed a critical threshold, p53 activation fails to produce oscillatory behaviour [11]. Besides, the p53 and its negative regulator MDM2 are expected to continuously rise to a lower level than in the high-damage (i.e., high-dose) case [11]. However, experimental validation of these predictions is still limited, necessitating further investigation into how p53 signalling responds to varying radiation doses. To address this, we focused on the temporal changes in p53 and MDM2 protein levels as well as on the transcriptional activation of p53-dependent genes, in human MCF-7 breast cancer cells following irradiation with different doses. The model system MCF-7 cells were irradiated with high, therapeutic, and low doses of 𝛾-radiation. Protein levels of p53 and MDM2 were measured at multiple time points using western blotting (WB). Concurrently, real-time quantitative PCR (RT-qPCR) was employed to quantify changes in the expression of *MDM2*, *CDKN1A* and growth arrest and DNA damage inducible alpha (*GADD45A*) as key p53 target genes involved in cell cycle regulation and apoptosis. By systematically tracking these molecular events, we aimed to determine whether low-dose radiation elicits a fundamentally different regulatory response compared to higher doses, enhancing our understanding of its cellular effects.

## Materials and methods

### Cell culture

The MCF-7 human breast cancer cell line was established in 1970 by Dr. Soule and colleagues at the Michigan Cancer Foundation, Detroit, from a pleural effusion of a 69-year-old Caucasian woman with metastatic adenocarcinoma (cat. n. HTB-22, ATCC) [20]. This cell line is widely used in in vitro breast cancer studies due to its characteristics resembling differentiated mammary epithelium. It has a typical doubling time of 30–40 h. The MCF-7 cell line expresses wild-type p53 and wild-type MDM2 and exhibits clear oscillatory behaviour of p53 in response to various types of irradiation, such as X-ray and 𝛾-radiation, as demonstrated in several studies [16, 18]. Notably, it is the first breast cancer cell line shown to be hormone responsive, making it highly valuable for experimental therapeutics research.

MCF-7 cells were cultured in tissue culture flasks at 37 °C and 5% CO_2_ in modified Dulbecco’s Eagle Medium 1X (DMEM, Gibco), supplemented with 10% fetal bovine serum (FBS, Sigma), 0.5% penicillin-streptomycin (P/S, Sigma), and 10 µg/mL bovine insulin (Sigma). During incubation, the cells were cultured as monolayers, reseeded before reaching 80% confluence.

### Sample irradiation

**Fig. 2:**
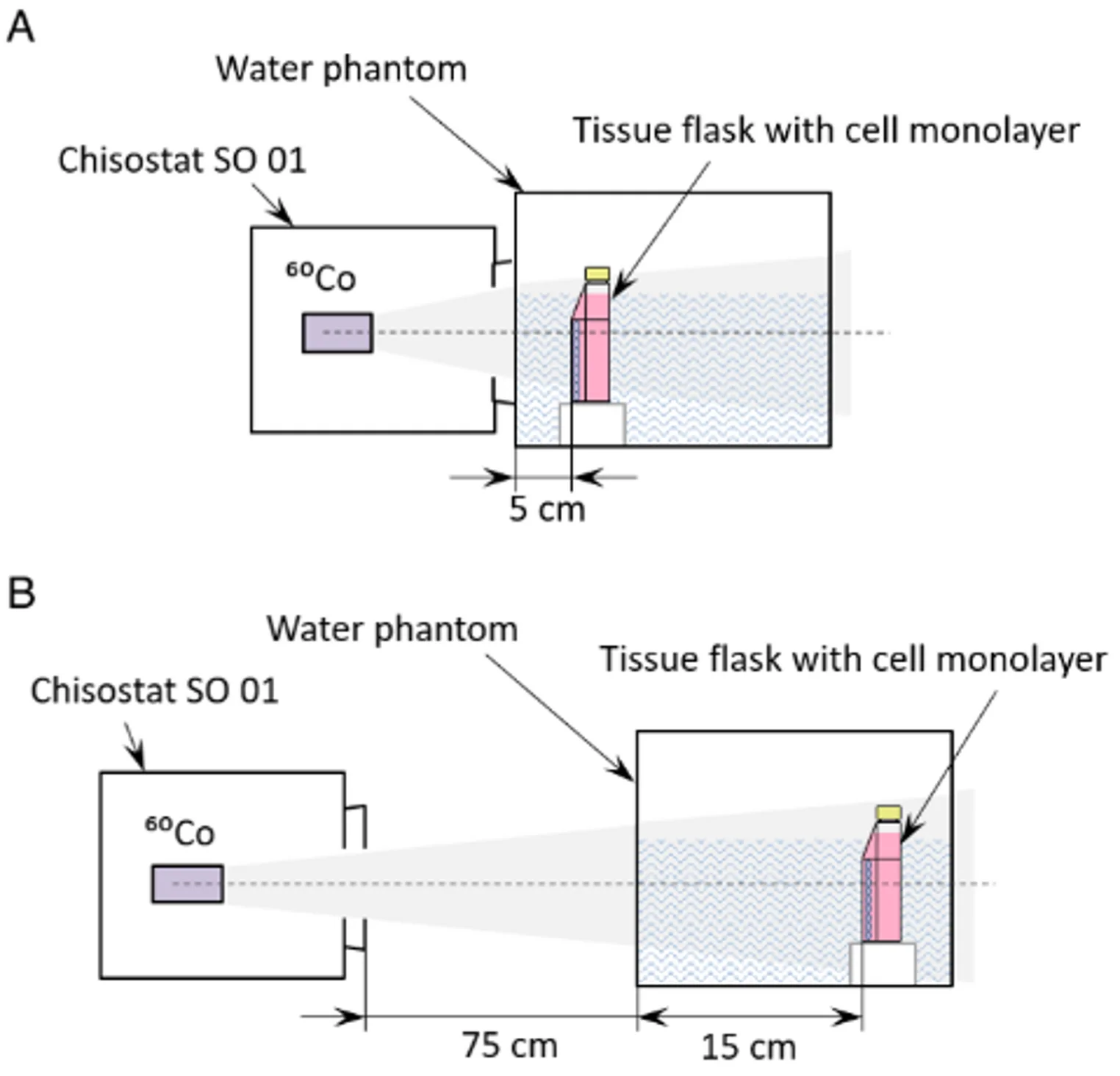
Scheme of the experimental setup of the cell irradiations in a water phantom by ^60^Co γ-rays at high (**A**) and low doses (**B**)

Samples were irradiated by ^60^Co γ-ray source (Chisostat SO 01 system) at the Authorized Metrology Center of the Nuclear Physics Institute of the Czech Academy of Sciences (Prague, Czech Republic). The experimental setup used for irradiation of MCF-7 cells is illustrated in Fig. 2. For high-dose exposures (8 and 2 Gy; panel A), cell cultures were irradiated at the dose rate of about 2.2 Gy/min, and the flask’s reference depth was equal to 5 cm in a water phantom. At low doses (1, 0.25 and 0.1 Gy; panel B), the dose rate was decreased to approximately 0.125 Gy/min, and the effective measurement point was adjusted to 75 cm in the air with an additional 15 cm depth in a water phantom. Here and throughout this work, unless stated differently, the term “high dose” refers to doses greater than 1 Gy, while “low dose” refers to doses of 1 Gy or lower, respectively. Due to the horizontal beam orientation, the cells were irradiated in a vertical position in T25 flasks fully filled with medium. The irradiation field was restricted to an area of 10 × 10 cm^2^ by a primary and secondary collimator. The dose calibration measurements were performed by a cylindrical PTW 30013 ionization chamber (PTW, Freiburg, Germany). The maximal positioning error was 2 mm, which corresponds to a dose uncertainty of less than 2% (calculated from the depth–dose curve).

The primary aim of this work was to extend the knowledge on p53 dynamics, extensively studied in the high-dose region, to low-dose irradiation. Therefore, the low-dose (1, 0.25 and 0.1 Gy) irradiations were performed with three independent repeats, while the 8 Gy irradiations were repeated with two biologically independent samples and the 2 Gy campaign was performed once just for confirmation purposes.

### Protein extraction and western blotting

Protein levels of p53, MDM2 and γ-H2AX were measured at multiple time points (0, 1, 2, 3, 4, 5, 6, 7, and 8 h) post-irradiation. Control samples (0 h) correspond to time post irradiation necessary for sample retrieval and transfer to the laboratory estimated as about 5–15 min. Flasks containing control or irradiated MCF-7 cells were washed twice with cold Dulbecco’s phosphate buffered saline (dPBS, pH 7.4, Sigma-Aldrich). To prepare protein extract, cells from each flask were detached using a cell scraper transferred to a 1.5 ml Eppendorf tube, and centrifuged for 3 min. After centrifugation, a sample buffer (SB) was added to the cell pellet. SB consisted of 62.5 mM Tris pH 6.8, 2% sodium dodecyl sulphate (SDS), 10% glycerol, 5% 2-mercaptoethanol, and 0.002% bromophenol blue (final concentrations). Each sample was heated at 95 °C for 5 min to completely denature the proteins, followed by 30 s of ultrasonication. Samples were stored at -80 °C.

Proteins were separated by molecular weight using gel electrophoresis. The separated proteins were transferred from the gel (4–15%, Bio-Rad) to polyvinylidene difluoride membranes (PVDF, Bio-Rad) using a transfer buffer, composed of 25 mM Tris, 192 mM glycine, 20% methanol, and distilled water (final pH 8.3). Membranes were subsequently blocked in a solution containing 5% non-fat milk. Following blocking, membranes were incubated overnight at 4 °C with the primary antibodies (rabbit polyclonal actin antibody, 1:1000, A2066, Sigma-Aldrich; mouse monoclonal p53 antibody, 1:1000, sc-126, Santa Cruz Biotechnology; mouse monoclonal MDM2 antibody, 1:500, OP-46, Millipore; mouse monoclonal Anti-phospho-Histone H2A.X (Ser139), clone JBW301, 1:500, Merck).

Following incubation, unbound primary antibodies were washed away with Tris-buffered saline containing 0.1% Tween-20 (TBST, Thermo Fisher Scientific). Secondary antibodies (Anti-Mouse IgG, 1:5000, 7076, Cell Signalling; Anti-Rabbit IgG, 1:6000, NA9340, Thermo Fisher Scientific) were added and incubated for 1 h at room temperature. After washing with TBST, the membranes were incubated with Chemiluminescent Substrate (Thermo Scientific) for 5 min. Each sample was analysed by western blot at least twice. The images were captured using the Uvitec Cambridge instrument.

### RNA extraction and RT-qPCR assay

For the RT-qPCR assay, two control samples were prepared for each experiment. The “0” sample was irradiated with the specified dose and harvested immediately afterward (within 5 min), while the sham-irradiated, “0_0”, sample was not irradiated and was harvested simultaneously with the “0” sample. The remaining irradiated samples were collected at the designated time points. Total RNA was isolated from MCF-7 cells using a PureLink RNA Mini Kit (Ambion) according to the manufacturer’s protocol. Two hundred nanograms of total RNA were reverse transcribed using random hexamer primers (Invitrogen) and M-MLV reverse transcriptase (Promega). cDNAs were amplified by the LightCycler^®^ 480 system (Roche) using the SYBR Green I master mix (Roche).

The following primers were used: 5′-AGCCACATCGCTCAGACAC-3′ and 5′-GCCCAATACGACCAAATCC-3′ for *GAPDH*; 5′-TTCTACCACTCCAAACGCCG-3′ and 5′-GGCAGAAGATGTAGAGCGGG-3′ for *CDKN1A*; 5′-CTGTGAGTGAGTGCAGAAAGC-3′ and 5′-CCAGCCGAGAATTCCTCCAAA-3′ for *GADD45A*; 5′-GATGTTGGGCCCTTCGTGAG-3′ and 5′-AGCCCTCT TCAGCTTGTGTTG-3′ for *MDM2*. All primers were designed using the Primer designing tool from the National Library of Medicine NIH [21] and DNASTAR Lasergene 13 SeqBuilder software [22]. All reactions were performed in triplicate. All mRNA levels were normalized to *GAPDH* mRNA. The data were plotted as a relative number to the average of the controls (“0_0” and “0” samples).

### Data analysis

Data are plotted as mean values and standard deviations from independent biological repeats (for 2 Gy irradiations, from technical replicates) as specified above.

To assess whether low-dose response of the p53 system qualitatively differs from that at high doses, the measured WB and RT-qPCR data normalized to controls as described above were analysed as follows. Individual datasets (i.e., separately for p53 and MDM2 WB and for *CDKN1A*, *GADD45A* and *MDM2* RT-qPCR dynamics) were fitted to damped oscillator response (y) to exponentially decreasing signal, namely.

1 $$\begin{aligned}y{\text{ }} &= 1 + H({t_0}) a_0 \times \\& \left[\exp ( - \alpha (t - {t_0})) - \exp ( - \zeta {\omega _0}(t - {t_0})) \times \left[\cos ({\omega _1} (t - {t_0})) + \frac{\zeta {\omega _0} - \alpha}{{\omega _1}} \sin ({\omega _1} (t - {t_0}))\right]\right] \end{aligned}$$

Here, ω_0_ denotes the system’s natural frequency, ζ the damping ratio, $\omega_1 = \omega_0 \sqrt{1 - {\zeta ^2}}$, α stands for the signal decay, a_0_ the amplitude of the response, H denotes Heaviside function (unit step, acting like an off/on switch: H(t < t_0_) = 0, H(t ≥ t_0_) = 1), t_0_ stands for the effective delay in response post (acute) irradiation.

The fitting was performed in R using the nlsLM function that employs Levenberg-Marquardt algorithm for nonlinear least squares fitting of data. The system’s natural frequency (ω_0_) was adjusted uniquely for all data, neglecting the differences among systems that span from initial radiation-induced damage to DNA over the triggered p53 to its diverse downstream targets. Likewise, the signal decay (α) was adjusted uniquely, assuming that the effective signal (DNA damage, but for downstream targets also including p53) decays mono-exponentially, identically for all endpoints. The remaining parameters (a_0_, t_0_, ζ) were fitted to each dynamics dataset (5 endpoints and 5 doses) independently. The data were weighted with the inverse square of their standard deviations (standard deviations falling below 0.001 were considered unrealistically low, and hence replaced by this value). Akaike information criterion (AIC) was used to evaluate the expected information loss if the damped oscillations were neglected, i.e. if the given model was replaced by a time-independent one (a constant) fitted to the data. AIC deals with the trade-off between models’ goodness of fit and simplicity, balancing the risks of over- and underfitting. Contrary to ANOVA-based tests, AIC is not limited to nested models but can compare any pair or multitude of models. Specifically, for a set of models with minimum of AIC values denoted by AIC_min_, the probability that the i-th model (with AIC_i_) minimizes the information loss can be estimated as exp((AIC_min_ − AIC_i_)/2) [23]. Herein, dataset-specific (a_0_, t_0_, ζ) as well as universal parameters (ω_0_, α) were counted in AIC calculated for each dataset, so that the judgements on the presence of damped oscillatory behaviour remain conservative.

## Results

### Radiation-induced γ-H2AX formation in MCF-7 cells

**Fig. 3:**
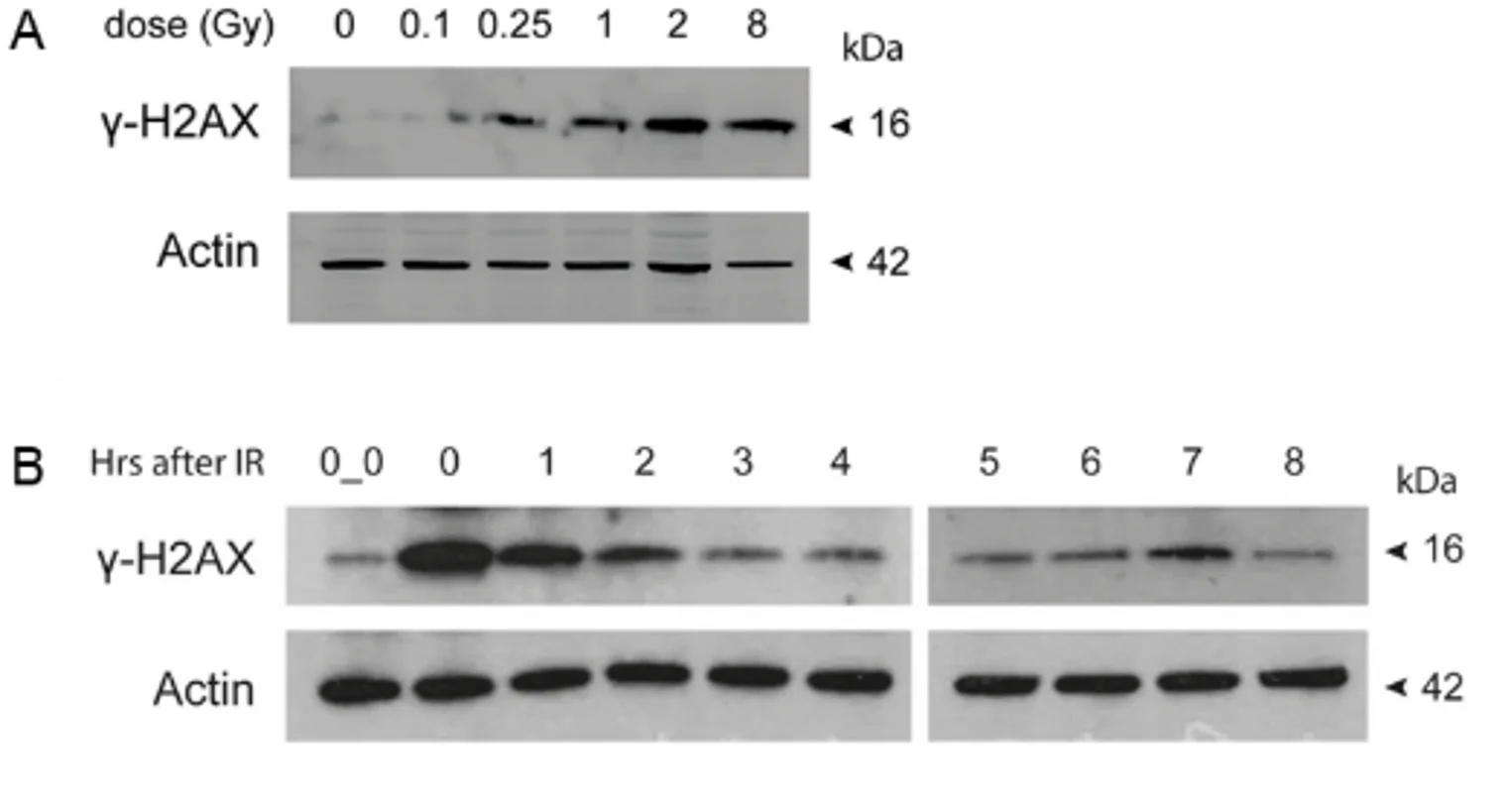
Radiation-induced γ-H2AX protein levels in MCF-7 cells after γ-radiation by doses 0, 0.1, 0.25, 1, 2, and 8 Gy immediately post irradiation (**A**) and time-course analysis of γ-H2AX protein level following exposure to 8 Gy (**B**). Actin served as a loading control

In the initial phase of the investigation, radiation-induced DNA damage has been assessed by γ-H2AX protein levels in irradiated samples. γ-H2AX were measured in MCF-7 cells after γ-radiation by doses 0, 0.1, 0.25, 1, 2, and 8 Gy, together with time-course analysis of γ-H2AX protein level following exposure to 8 Gy (Fig. 3). As shown in Fig. 3A, γ-H2AX levels increased in a dose-dependent manner immediately after irradiation. While only a modest increase was observed following exposure to 0.1 Gy, a more pronounced accumulation of γ-H2AX was detected at doses of 0.25 Gy and above. These results confirm successful induction of DNA double-strand breaks across the investigated dose range. The temporal dynamics of γ-H2AX following 8 Gy irradiation is shown in Fig. 3B. γ-H2AX levels increased rapidly after irradiation, reached maximal levels within the first 1–2 h and gradually declined over time.

### Ionizing radiation triggers oscillations of p53 at higher doses

**Fig. 4:**
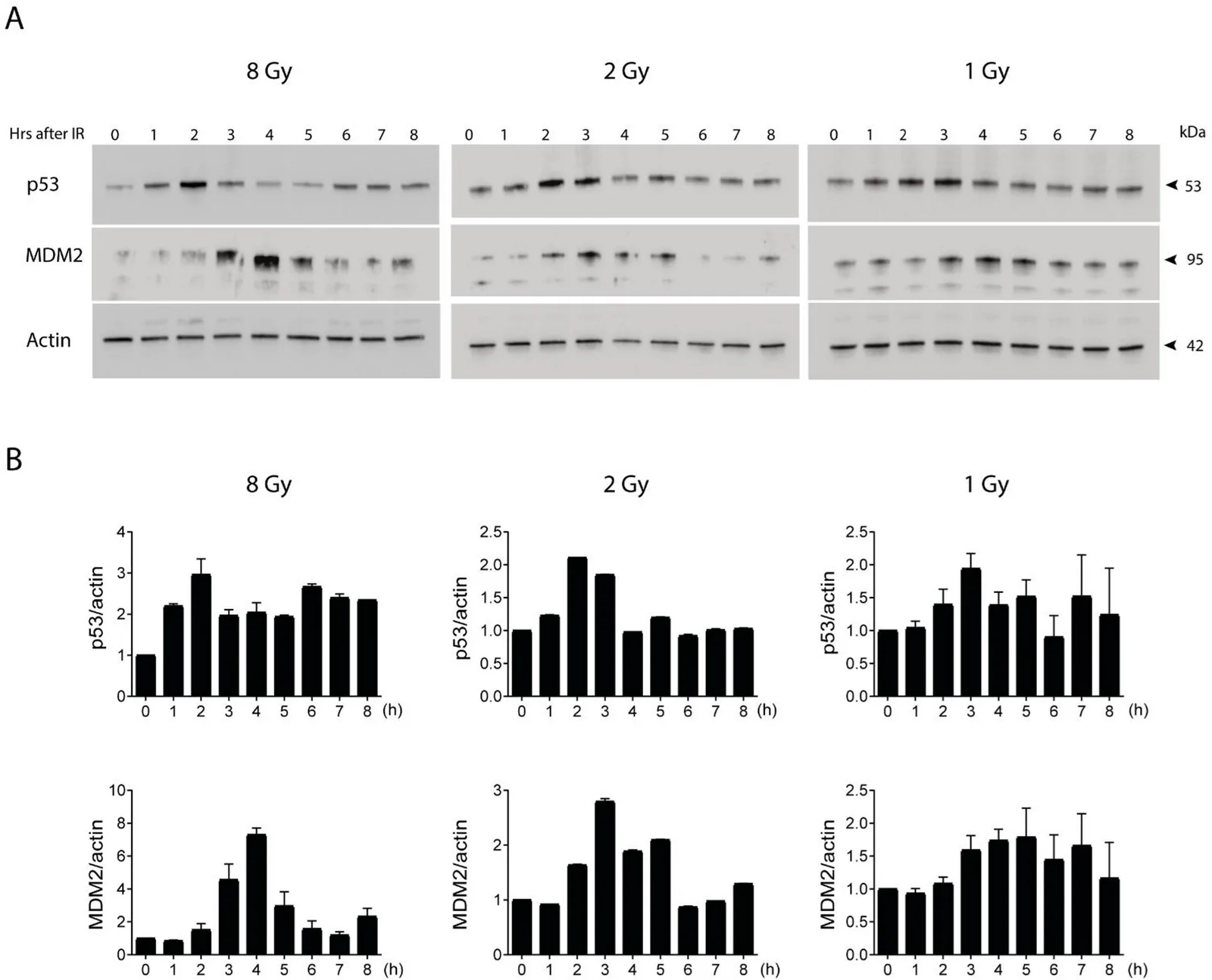
Levels of p53 and MDM2 proteins after γ-radiation by 8, 2, and 1 Gy assessed by western blotting (**A**). Quantification relative to the actin loading control (**B**). Error bars represent standard deviations from the biological repeats; standard deviations from technical replicates were used for 2 Gy irradiations

Subsequently, the relative levels of p53 and MDM2 proteins were measured in MCF-7 cells exposed to 8, 2, and 1 Gy of γ-radiation in a water phantom. The western blot results are shown in Fig. 4.

While actin levels remained virtually constant across the doses and time points, the levels of p53 and MDM2 notably varied, being enhanced by up to 3 times for p53 at 2 h or up to 8 times for MDM2 at 4 h post 8 Gy exposure, relative to controls. The analysis indicates that the outcomes of γ-irradiation at 8, 2, and 1 Gy are consistent with previously published studies [16, 18]. Ionizing radiation triggers oscillations of p53 in MCF-7 cells, with a stable oscillation period of approximately 4 h. MDM2 exhibits a similar oscillation pattern, delayed by about 2 h relative to p53.

**Fig. 5:**
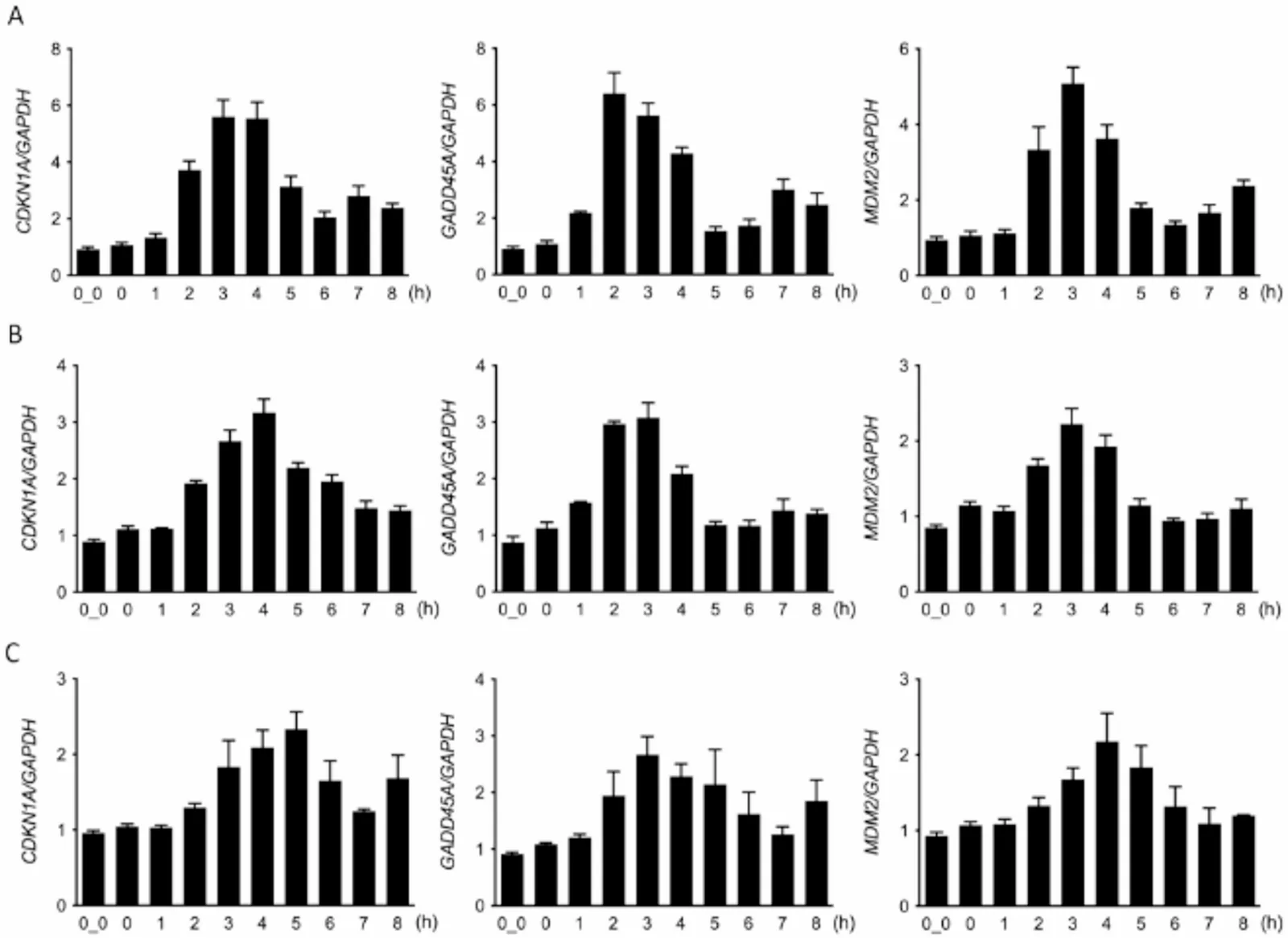
Real-time qPCR analysis of mRNA levels of *CDKN1A*, *GADD45A*, and *MDM2* after γ-radiation by 8 Gy (**A**), 2 Gy **(B**) and 1 Gy (**C**). *GAPDH* was used as a reference gene. Error bars represent standard deviations from the biological repeats; standard deviations from technical replicates were used for 2 Gy irradiations

To investigate how p53 dynamics control gene expression, we selected three well-characterized p53 target genes representing different functional pathways. This group included genes involved in cell cycle arrest and DNA repair *CDKN1A*, and *GADD45A*, as well as *MDM2*, which regulates p53 levels. The RT-qPCR results for these genes are shown in Fig. 5. Notably, the subset of genes displayed a clear positive response that reflects the dynamics of p53 protein levels. At 8 Gy, the expression level of all three genes was upregulated by a factor of 6 at 2–3 h post irradiation. With decreasing dose, the factor was reduced to about 3 at 2 Gy and about 2 at 1 Gy and the maximum shifted to 1–2 h later time points.

### Transient peak of p53 at lower doses of irradiation

**Fig. 6:**
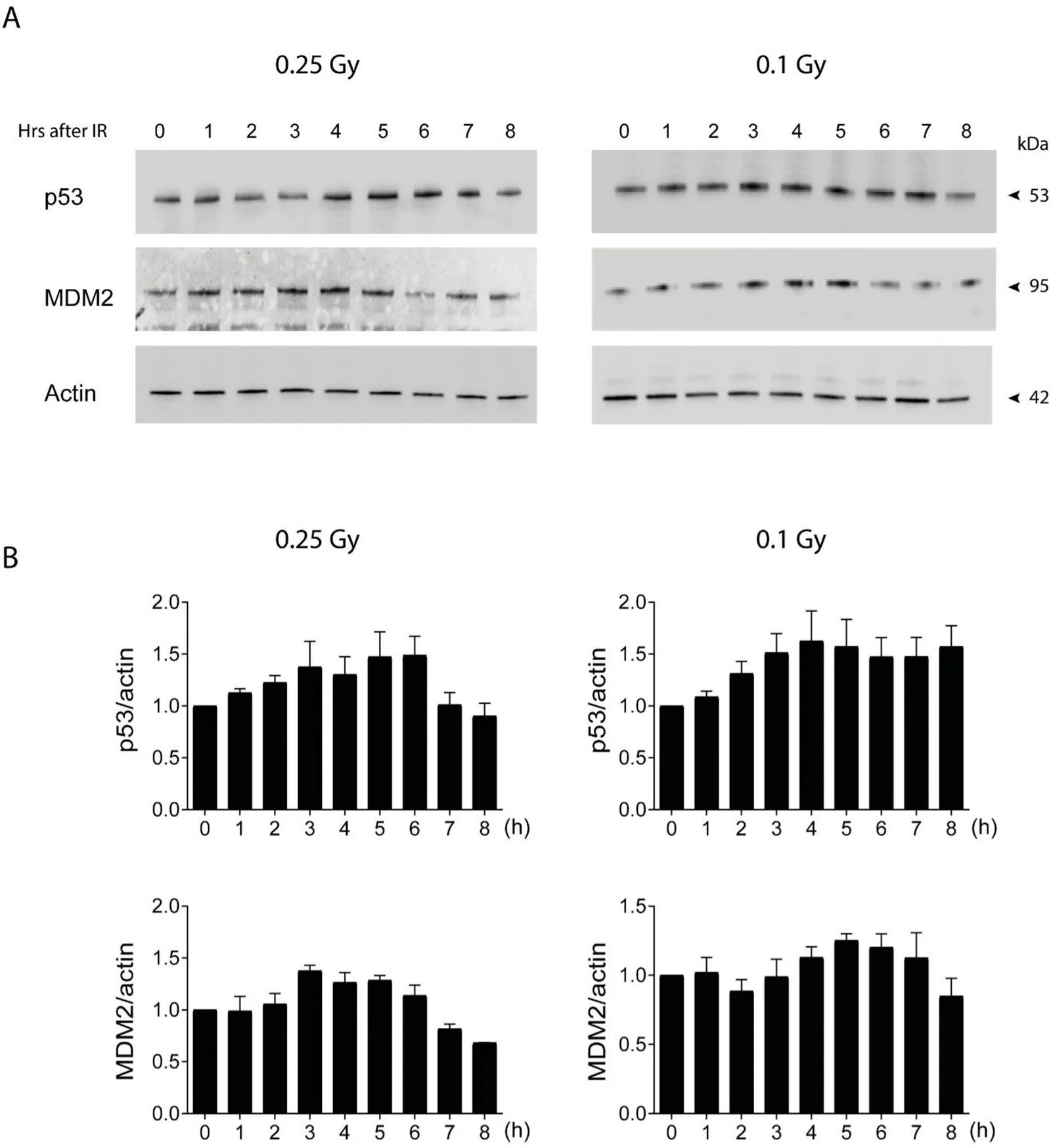
Levels of p53 and MDM2 proteins after γ-radiation by 0.1 and 0.25 Gy confirmed by western blot (**A**). Quantification relative to the actin loading control (**B**). Error bars represent the standard error of the mean of 3 independent experiments

Based on classical mathematical predictions, low levels of cellular stress may be insufficient to trigger oscillatory p53 dynamics, resulting in a weaker p53-MDM2 response than that observed following high levels of DNA damage. To investigate whether transient p53 and MDM2 responses can be detected at low radiation doses, we examined the temporal expression dynamics of both proteins in MCF-7 cells to low doses of γ-irradiation. Transient peaks in both p53 and MDM2 expression were detected following irradiation with doses as low as 0.25 and 0.1 Gy (Fig. 6), indicating that dynamic changes in these proteins can be observed even after low-dose irradiation. The upregulation of protein levels reaches factors 1.2 to 1.5 at 3 to 6 h post irradiation, followed by decline.

**Fig. 7:**
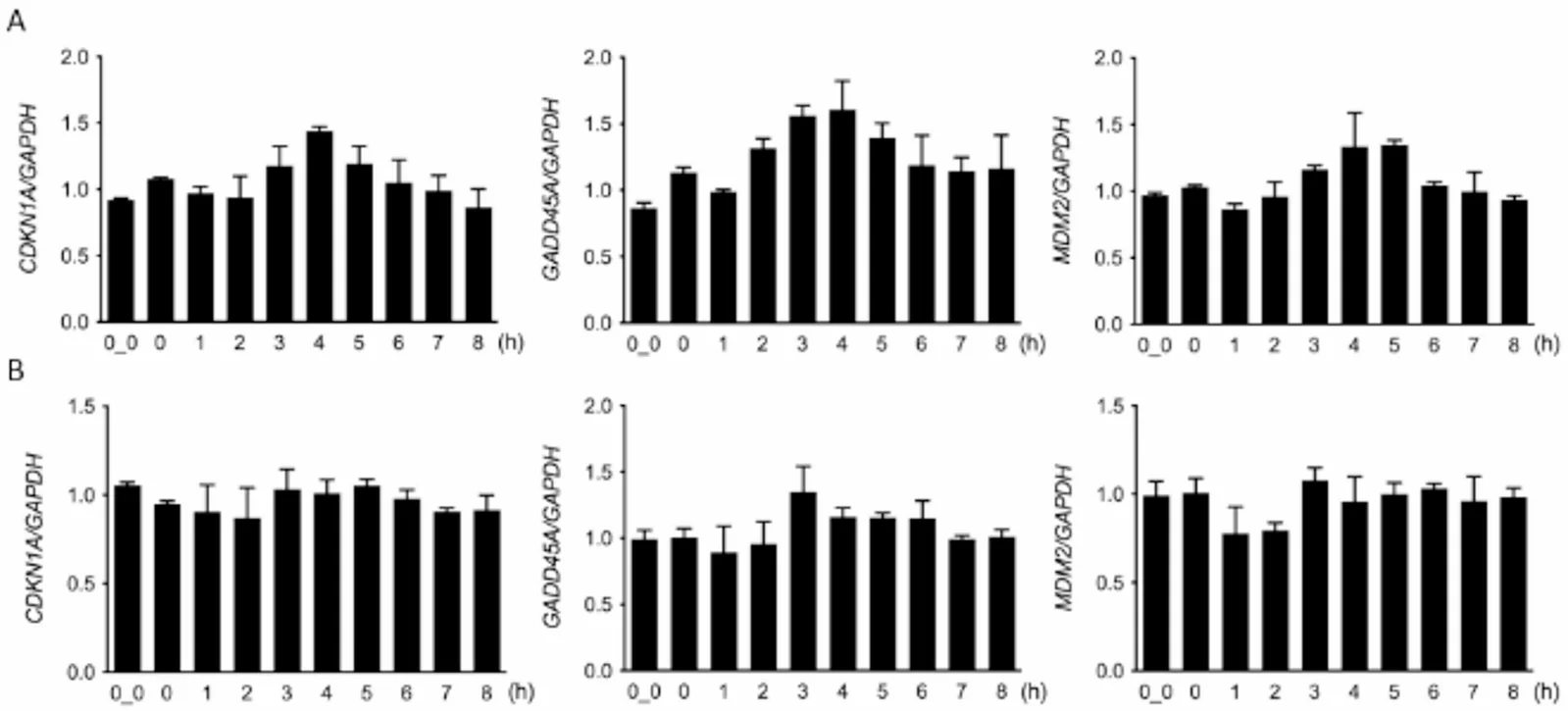
Real-time qPCR analysis of mRNA levels of *CDKN1A*, *GADD45A*, and *MDM2* after γ-radiation at 0.25 Gy (**A**) and 0.1 Gy (**B**). *GAPDH* was used as a reference gene. Error bars represent the standard error of the mean of 3 independent experiments

In addition to western blot analysis, we also investigated the expression of p53 target genes using quantitative RT-qPCR analysis. As shown in Fig. 7, the RT-qPCR results for *CDKN1A*, *GADD45A*, and *MDM2* expression revealed a transient peak expression pattern, mirroring the fluctuations in p53 and MDM2 protein levels, even at low irradiation doses of 0.25 Gy and 0.1 Gy. Again, the upregulation factors were as high as 1.5 observed at about 4 h post irradiation by 0.25 Gy. At 0.1 Gy, the results suffer from large scatter, with the highest upregulation of 1.3 seen for *GADD45A* 3 h post irradiation.

**Fig. 8:**
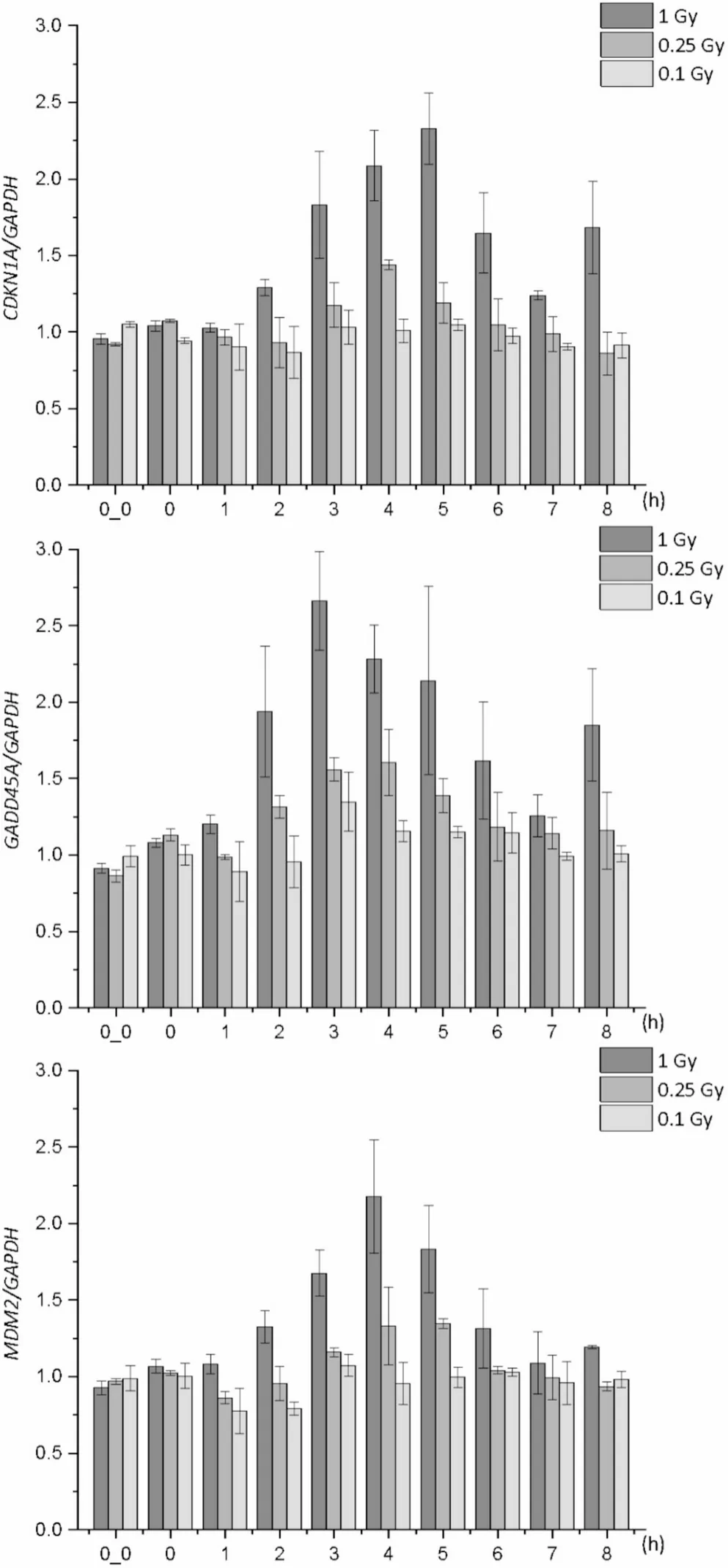
Relative expression of mRNA levels of *CDKN1A*, *GADD45A*, and *MDM2* after γ-radiation determined by quantitative RT-qPCR analysis after γ-radiation at 1, 0.25, and 0.1 Gy. Error bars represent one standard error of the mean of 3 independent experiments

The RT-qPCR analysis of p53-responsive gene expression following irradiation with doses of 1, 0.25 and 0.1 Gy is summarized in Fig. 8, where the three doses are shown side by side to facilitate direct comparison of the responses. As shown, *CDKN1A* exhibits a peak at 3–5 h for the three doses studied. A similar pattern is observed also for *GADD45A* and *MDM2*.

### p53 related dynamics are consistent with damped oscillatory behaviour

**Fig. 9:**
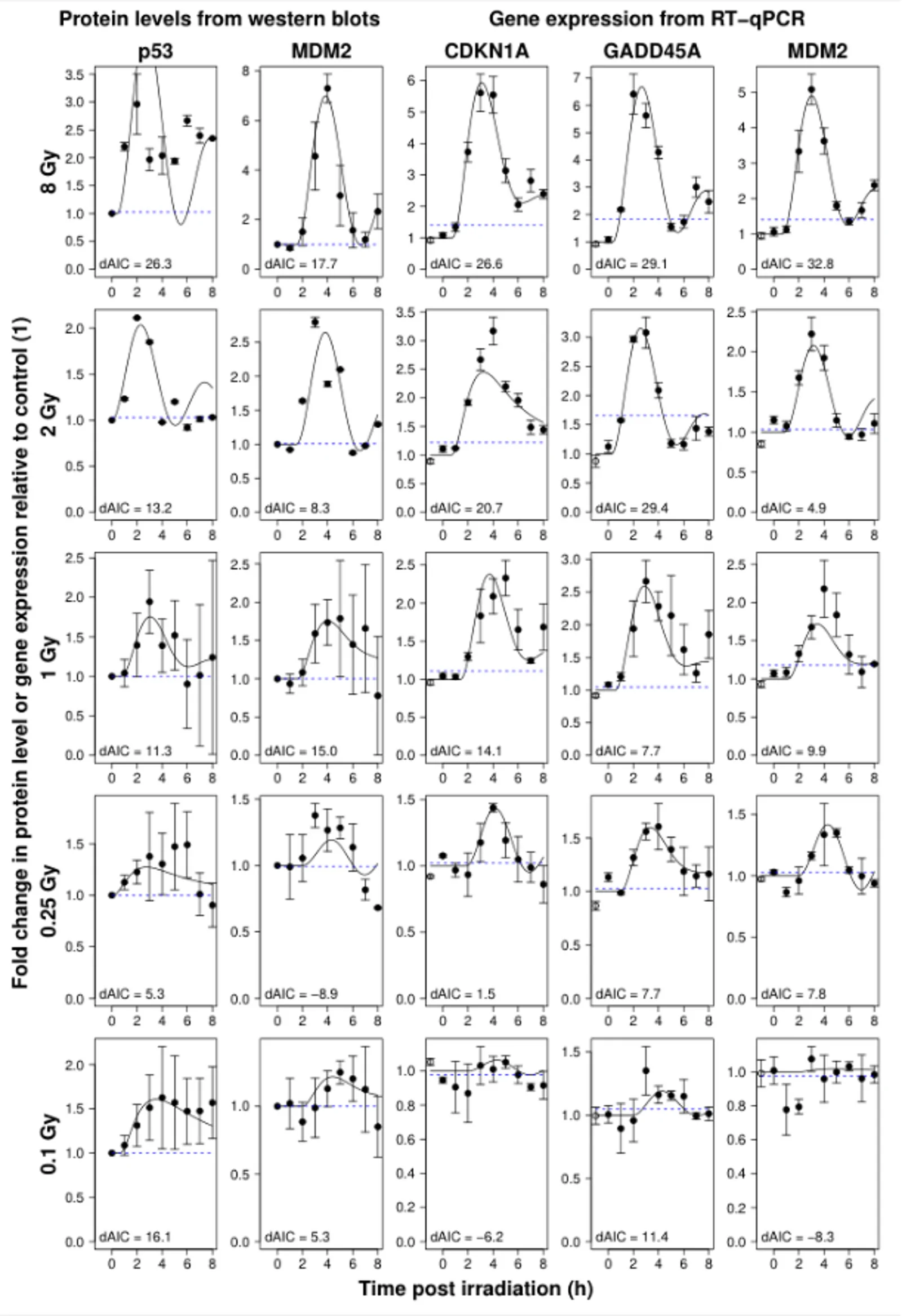
Quantitative analysis of individual experimental datasets on the dynamics of protein levels of p53 and MDM2 from western blots (WB p53, WB MDM2) and *CDKN1A*, *GADD45A* and *MDM2* gene expression from RT-qPCR (symbols) by a damped oscillator model (solid black lines). Sham-irradiated controls in RT-qPCR (empty symbols) are plotted at the time point of -1 h. Also shown are fits using a constant model (dashed blue lines). Positive values of dAIC denote the damped oscillatory model being preferred over the constant one, negative values the opposite case

To test whether low-dose dynamics conceptually differs from the oscillatory high-dose one, a quantitative analysis of the measured data was performed. The results are presented in Fig. 9. For all endpoints and doses studied, the dynamics revealed by the data (symbols, uncertainties depicted by error bars) was analysed using a damped oscillatory model (solid black lines) or alternatively a constant model (dashed blue lines). The numbers in the bottom-left part of each panel are differences in AIC values between the two models, dAIC = AIC_const_ - AIC_osc_; they characterize the appropriateness of the damped oscillatory model relative to that of a constant, with increasing values suggesting increasingly favourable performance of the damped oscillatory model (positive value: damped oscillatory model preferred; negative value: constant model preferred). Specifically, e.g. for p53 protein at 0.1 Gy or for *CDKN1A* gene at 0.25 Gy, the constant model is exp(-16.1/2) ~ 0.0003 or exp(-1.5/2) ~ 0.5 times as probable as the damped oscillatory model to minimize the information loss, respectively. For MDM2 protein at 0.25 Gy, the damped oscillatory model is less probable than the constant model (by a factor of exp(-8.9/2) ~ 0.01), despite fitting the data reasonably; this case illustrates the penalization for the number of model parameters embedded in AIC. Taken together, the present data provide insufficient support for the damped oscillatory model (i.e., the constant model is preferred) for three datasets (*MDM2* and *CDKN1A* genes at 0.1 Gy and MDM2 protein at 0.25 Gy), the two models perform about comparably for one dataset (*CDKN1A* gene at 0.25 Gy), and in the remaining 21 cases the damped oscillatory model is clearly preferred. Closer inspection of the model parameters (Online Resource 1) reveals that amplitudes a_0_ of the response, while exhibiting some scatter among endpoints, increase with increasing dose, close to directly proportionally. This is in agreement with the linear relationship between radiation-induced DNA damage and the applied dose reported previously [24–26]. Further, the delay t_0_ in the onset of damped oscillations exhibits a decreasing trend with increasing dose, as might have been expected. The damping ratio 𝜁 increases with decreasing dose, meaning that the period of damped oscillations increases or they even diminish as the system moves towards a critically damped or overdamped one (𝜁 approaching or exceeding unity, respectively). Since the data were gathered over a limited time interval (0–8 h) only and the data points possess rather large error bars as is common in biology, the derived parameters of damped oscillations suffer from large uncertainties. Yet, the performed analysis demonstrates that across the studied doses, including doses as low as 0.1 Gy, the observed protein and mRNA dynamics are consistent with damped oscillatory behaviour known for the p53 system at high doses.

## Discussion

The p53 signalling axis is a central determinant of cellular response to ionizing radiation, orchestrating DNA repair, cell cycle arrest, and apoptosis downstream of radiation-induced DNA damage [1, 2]. In particular, it sheds light on signalling pathways linked to the p53 protein, which is regulated via a negative feedback loop involving its transcriptional target, MDM2 [11, 13]. DNA damage repair, including the repair of double-strand breaks (DSBs), can be detected at doses as low as 0.1 Gy [27], with the number of induced breaks increasing linearly with radiation dose [28]. At these doses, the formation of DSB markers, such as γ-H2AX foci, signals the activation of repair mechanisms [29]. The efficiency of DNA repair is closely related to cellular radiosensitivity, with more radiosensitive cells generally exhibiting less effective repair mechanisms [30, 31].

Understanding the dose-dependent activation of p53 is essential for defining the minimal radiation thresholds that trigger cellular protective mechanisms, ultimately determining cell fate by promoting either survival or apoptosis and thereby influencing cellular radioresistance and radiosensitivity [32, 33]. Therefore, investigating the effect of low-dose radiation is essential for understanding the molecular mechanisms underlying cell radiosensitivity.

In this study, the MCF-7 cell line, a well-established model of hormone-dependent breast cancer, was used to investigate the activation dynamics of the p53 protein. To experimentally verify the induction of DNA damage following irradiation, γ-H2AX protein levels were assessed by western blot analysis (Fig. 3). The observed increase in γ-H2AX across the applied dose range provides evidence that the irradiation protocol effectively triggered the DNA damage response. The rapid appearance of γ-H2AX followed by its subsequent decline is consistent with the transient nature of this marker and may reflect the progression from damage recognition towards repair of radiation-induced DNA lesions.

We examined the dose-dependent regulation of p53 and MDM2 in MCF-7 cells, focusing on whether p53 induction persists at low radiation doses. To validate the experimental protocol, the initial phase involved irradiating the cells with high doses of γ-radiation to ensure methodological reproducibility and reliability. The results obtained (Fig. 4) were consistent with previously published data [11, 16, 18]. Ionizing radiation triggers enhanced expression of p53 in MCF-7 cells, with a transient peak at approximately 2 h. MDM2 exhibits a similar pattern, delayed by about 2 h relative to p53.

Beyond assessing changes in p53 and MDM2 protein levels after high-dose irradiation, the expression of p53-responsive genes was analysed using real-time quantitative PCR. The selected target genes included *CDKN1A* and *GADD45A*, which mediate cell cycle arrest, as well as *MDM2*, the primary regulator of intracellular p53 levels. As shown in Fig. 5, the expression of these genes increases 2–3 h after high-dose γ-radiation, exhibiting a distinct response to radiation. This pattern further corroborates the expression dynamics of p53 protein levels.

Given the biological significance and potential risks associated with low-dose radiation, the dose range of 0 to 1 Gy represents a critical region for understanding cellular responses to radiation exposure. Mathematical models have shown that activation of p53 induces an oscillatory pattern of p53 and MDM2 after high-dose irradiation [11, 34], but when the stress signal falls below a critical threshold (at low doses), activation of p53 no longer leads to oscillatory behaviour [11]. The present data indicate that the p53-MDM2 regulatory network remains dynamically responsive even under low levels of radiation-induced stress (Fig. 6). The detection of transient p53 and MDM2 responses at doses as low as 0.1 Gy suggests that the threshold for dynamic activation of this pathway may be lower than predicted by classical models. Rather than being absent at low doses, the response appears to be progressively attenuated as the radiation-induced stress decreases, indicating that the p53 system can preserve its dynamic character even when the initiating signal is relatively weak. This is further supported by the response of p53 downstream target genes (Fig. 7).

These qualitative conclusions are supported by the performed mechanistic analysis (Fig. 9 and Online Resource 1). Here, all 5 endpoints (p53 and MDM2 proteins assessed by western blotting, and *CDKN1A*, *GADD45A* and *MDM2* genes assessed by RT-qPCR) were assumed to respond to the same signal that decreases exponentially with time. This simplification is motivated by the fact that the primary stress signal is radiation-induced DNA damage, whose biphasic repair was approximated by a single exponential decay over the studied period of 8 h post irradiation. Further, all 5 endpoints were assumed to belong to the same system, characterised by a unique intrinsic oscillation frequency fitted commonly to the whole dataset. This approximation somewhat neglects the differences among the systems, in particular the involvement of additional steps leading from p53 to its downstream targets, which were accounted for in a simplified manner only, through the delay parameters t_0_. Despite these limitations, the performed quantitative analysis strongly suggests that there is no qualitative difference in p53 dynamics triggered by low vs. high doses; merely the upregulation factors are lower and the onset delayed by low vs. high doses.

The reported findings provide a foundation for further investigation using high-resolution imaging techniques, such as confocal and fluorescence microscopy, to monitor p53 dynamics at the single-cell level. Additionally, epigenetic analysis, including ChIP-sequencing and single-cell RNA sequencing (scRNA-seq), offer promising approaches for identifying regulatory elements and refining the transcriptional responses regulated by p53. These methodologies will further enhance our understanding of p53 dynamics and its interaction with MDM2, paving the way for improved radiotherapy strategies and optimized irradiation protocols for hormone-dependent tumours.

## Conclusion

Dose- and time-dependent modulation of p53 and MDM2 protein expression and p53-regulated genes *CDKN1A*, *GADD45A*, and *MDM2* was studied in MCF-7 breast cancer cells exposed to ^60^Co γ-radiation. High doses (> 1 Gy) induced an early peak at approximately two hours post irradiation, followed by a decline and in some cases further oscillations across the studied period of 8 h, whereas lower doses (0.1–1 Gy) resulted in a delayed and attenuated peak. Except a few cases at 0.25 and 0.1 Gy where the peak amplitude was too small, the studied protein levels and gene expressions across all dose levels were consistent with damped oscillatory behaviour reported following high-dose irradiation previously. No clear qualitative difference from high-dose effects was observed for p53 signalling in response to doses of as low as 100 mGy.

## Supplementary Information

Below is the link to the electronic supplementary material.

Supplementary Material 1

## Data Availability

The datasets generated and analysed during the current study are available from the corresponding author on reasonable request.
