# Supplementary material for: Transient peak of p53 induction persists at low radiation doses in MCF-7 cells
Source: Mol Biol Rep. 2026 Oct 1;53(1):1668. doi: 10.1007/s11033-026-12787-7 (PMC13630799; doi:10.1007/s11033-026-12787-7)
Supplement: Supplementary file 1 [file 11033_2026_12787_MOESM1_ESM.pdf]

Supplementary Information

Transient peak of p53 induction persists at low radiation doses in MCF-7 cells

Irina Danilova[1,2], Jana Klementová[2,3], Šárka Jarošová[3,4], Pavel Kundrát[2], Martina Zíková[2,3], Marie Davídková[2*]

[1] Czech Technical University in Prague, Faculty of Nuclear Sciences and Physical Engineering, Břehová 78/7, 110 00 Praha 1, Czech Republic
[2] Nuclear Physics Institute of the Czech Academy of Sciences, Department of Radiation Dosimetry, Na Truhlářce 39/64, 180 00 Praha 8, Czech Republic
[3] Institute of Molecular Genetics of the Czech Academy of Sciences, Vídeňská 1083, 142 20 Prague 4, Czech Republic
[4] Charles University, Faculty of Science, Albertov 6, 128 00, Prague 2, Czech Republic

[*]Correspondence: davidkova@ujf.cas.cz

Journal: *Molecular Biology Reports*

This Supplementary Information provides additional details on the performed model analysis of dose-dependent dynamics of p53 and MDM2 protein levels, and *CDKN1A*, *GADD45A* and *MDM2* gene expression following acute 0.1 – 8 Gy γ-irradiation. The measured WB and RT-qPCR data normalized to controls were fitted to damped oscillator response to exponentially decreasing signal, Eq. (1) in the main text, with the following parameters: natural frequency ω_0, damping ratio ζ, signal decay α, amplitude of the response a_0, effective delay in response post (acute) irradiation t_0. The fitting was performed in R using the nlsLM function that employs Levenberg-Marquardt algorithm for non-linear least squares fitting of data.

Judgement of model quality was based on Akaike information criterion (AIC). AIC assesses how well a model matches the data using log-likelihood (which means residual sum of squares for the least square method), and adds a penalty for adding extra variables or parameters. It effectively deals with the trade-off between models' goodness of fit and simplicity, and balances the risks of over- and underfitting. Contrary to ANOVA-based tests, AIC is not limited to nested models but can compare any pair or multitude of models. Out of the tested set, the preferred model is the one with the lowest AIC. Specifically, for a set of models with minimum of AIC values denoted by AIC_{min}, the probability that the i-th model (with AIC_i) minimizes the information loss can be estimated as $\exp((AIC_{min} - AIC_i)/2)$.

Herein, AIC was used to evaluate the expected information loss if the damped oscillations were neglected, i.e. if the given model was replaced by a time-independent one (a constant) fitted to the data. Differences in AIC values between the two models, $dAIC = AIC_{const} - AIC_{osc}$, were used to characterize the appropriateness of the damped oscillatory model relative to that of a constant. Positive values of dAIC mean that the damped oscillatory model is preferred, while negative values imply that the constant model is preferred.

The system's natural frequency ω_0 was adjusted uniquely for all data, neglecting the differences among systems that span from initial radiation-induced damage to DNA to the triggered p53 or further to MDM2 or to diverse downstream targets of p53. Likewise, the signal decay α was adjusted uniquely, assuming that the effective signal (DNA damage, but for MDM2 and downstream targets also including p53) decays mono-exponentially, identically for all endpoints. These common parameters were adjusted manually, maximizing the sum of dAIC over the 25 datasets (5 endpoints: p53 and MDM2 protein levels, and *CDKN1A*, *GADD45A* and *MDM2* gene expression, each studied following 5 dose levels, 0.1, 0.25, 1, 2 and 8 Gy) with other parameters allowed to vary for individual datasets independently. This resulted in natural

frequency estimated as ω_0 = 1.25 h^{-1} (i.e., period $T_0 = 2\pi/\omega_0$ = 5 h) and signal decay as α = 0.2 h^{-1} (i.e., characteristic signal lifetime $T_\alpha = \alpha^{-1}$ = 5 h).

Then, these values were fixed via using them as both the upper and lower limits to the parameter values, while the remaining parameters (a_0, t_0, ζ) were fitted to each dataset independently (assuming plausible lower and upper limits such as $0.01 < \zeta < 0.99$ or $0 < t_0 < 2$ h). The data were weighted with the inverse square of their standard deviations (standard deviations falling below 0.001 were considered unrealistically low, and hence replaced by this value). The R's nlsLM function provided not only estimates of best-fitting parameter values but also their uncertainty, as well as the AIC calculated for each dataset (fitted alternatively with the damped oscillatory or constant model). Herein, dataset-specific (a_0, t_0, ζ) as well as universal parameters (ω_0, α) were counted as parameters in AIC calculated for each dataset, i.e. counting ω_0 and α not just once but for each dataset separately, so that the judgements on the presence of damped oscillatory behaviour remain conservative.

The estimated best-fitting parameter values and their uncertainties are listed in Table S1. Since the data were gathered over a limited time interval (0-8 h) only and the data points possess rather large error bars as is common in biology, the derived parameters of damped oscillations suffer from large uncertainties.

The derived parameters for the two proteins and three genes studied are plotted in Figure S1 (symbols) in dependence on the applied dose. There is a general trend of amplitudes a_0 of the response (panel A) decreasing with decreasing dose; indeed, the fitted values are rather well aligned (with coefficient of determination R^2 = 0.89) along the black line that shows the proportionality $a_0 = D * 0.54\ Gy^{-1}$ (note that a log scale was used, hence the proportionality line is not straight). The large uncertainties of the fitted values (not depicted to avoid an overly busy figure) prevent assigning specific trends to individual proteins and genes studied.

Angular frequency $\omega = \omega_0(1-\zeta^2)^{1/2}$ (panel B) and period of damped oscillations $T = 2\pi/\omega$ (panel C; periods > 10 h not plotted) exhibit notably larger scatter, with some tendency towards lower frequencies i.e. longer periods of oscillations as the radiation dose decreases.

Damping ratios ζ (panel D) exhibit even larger scatter, from largely undamped ($\zeta \sim 0$) to critically damped oscillations ($\zeta \sim 1$). There is a slight tendency towards stronger damping with decreasing dose.

Also the delayed onset t_0 (panel E) suffers from large scatter. Nevertheless, the p53 protein (green empty squares) shows the most rapid response to the radiation-induced damage, with delay times of 0 – 45 min. The enhanced expression of p53 target genes *GADD45A*, *MDM2* and *CDKN1A* is somewhat delayed post p53 protein, and the MDM2 protein (black triangles) is further delayed compared to the *MDM2* gene, so that the MDM2 protein follows the upstream p53 with a delay of about 1 – 2 hours. Note that these functional dependencies and trends were not assumed by the simplistic fitting model used herein but followed from the data. For more reliable quantitative conclusions on the damped oscillatory behaviour, its period, damping ratio etc., the signalling system would have to be followed for longer periods of time.

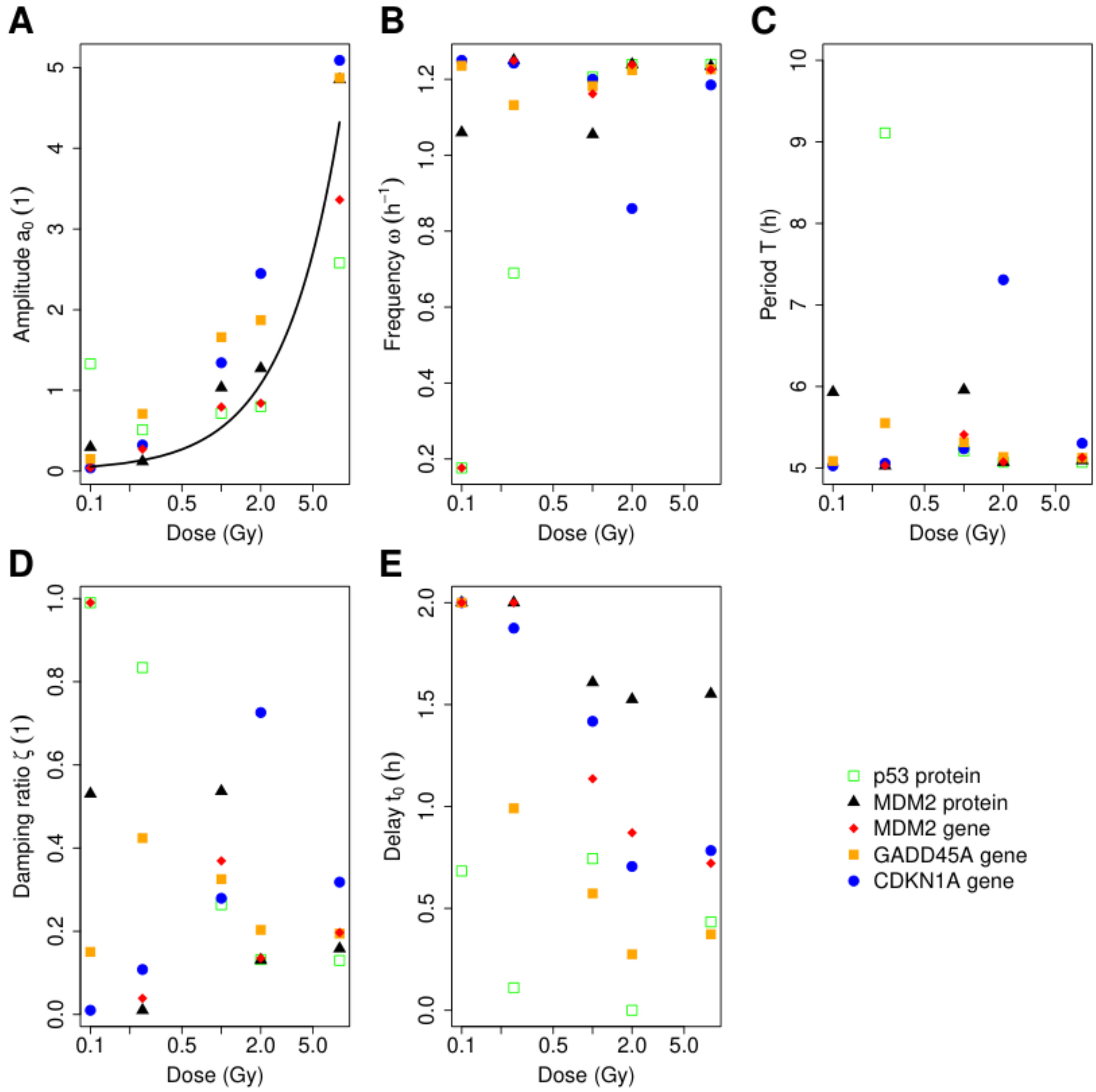

Figure S1: Best-fitting parameters (diverse symbols) derived from dose-dependent dynamics of p53 and MDM2 proteins and p53 target genes *GADD45A, MDM2 and CDKN1A* post γ-irradiation. Panel A: amplitude a_0 (black line: linear fit), panel B: angular frequency ω_0, panel C: period T, panel D: damping ratio ζ, panel E: delay t_0 of damped oscillations.

Table S1: Best-fit values of model parameters and estimates of their uncertainty (standard errors, SE).

	Dose D (Gy)	Amplitude a_0 (1)		Natural frequency ω_0 (h^{-1})		Damping ratio ζ (1)		Signal decay rate α (h^{-1})		Delay t_0 (h)	
		value	SE	value	SE	value	SE	value	SE	value	SE
p53 protein	8	2.58	2.98	1.25	0.28	0.13	0.12	0.2	0.19	0.43	0.74
	2	0.80	0.37	1.25	0.30	0.13	0.14	0.2	0.22	0	0.72
	1	0.72	0.76	1.25	0.33	0.26	0.43	0.2	0.36	0.74	0.45
	0.25	0.51	4.40	1.25	7.83	0.83	8.49	0.2	1.37	0.11	5.25
	0.1	1.33	5.57	1.25	1.13	0.99	2.70	0.2	0.60	0.68	0.44
MDM2 protein	8	4.85	2.35	1.25	0.15	0.16	0.15	0.2	0.19	1.55	0.35
	2	1.27	1.04	1.25	0.21	0.13	0.17	0.2	0.25	1.53	0.61
	1	1.03	2.40	1.25	0.66	0.54	1.16	0.2	0.56	1.61	0.43
	0.25	0.12	9.31	1.25	14.6	0.01	19.3	0.2	37	2	52
	0.1	0.30	2.46	1.25	3.04	0.53	4.34	0.2	2.04	2	4.36
MDM2 gene	8	3.36	0.41	1.25	0.03	0.20	0.02	0.2	0.03	0.72	0.07
	2	0.84	0.64	1.25	0.25	0.14	0.16	0.2	0.24	0.87	0.67
	1	0.80	0.74	1.25	0.91	0.37	0.61	0.2	0.22	1.14	0.91
	0.25	0.27	0.93	1.25	0.53	0.04	1	0.2	1.34	2	1.74
	0.1	0.04	15.9	1.25	348	0.99	397	0.2	62	2	260
GADD45A gene	8	4.87	1.04	1.25	0.06	0.19	0.05	0.2	0.06	0.37	0.09
	2	1.87	0.23	1.25	0.07	0.20	0.04	0.2	0.04	0.27	0.07
	1	1.66	1.78	1.25	0.43	0.33	0.53	0.2	0.25	0.57	0.35
	0.25	0.71	2.11	1.25	1.38	0.42	1.65	0.2	0.79	0.99	1.67
	0.1	0.15	0.26	1.25	0.76	0.15	0.53	0.2	0.78	2	2.15
CDKN1A gene	8	5.09	0.86	1.25	0.12	0.32	0.06	0.2	0.04	0.78	0.15
	2	2.45	2.96	1.25	0.53	0.73	0.99	0.2	0.21	0.71	0.18
	1	1.34	1.44	1.25	0.29	0.28	0.41	0.2	0.34	1.42	0.36
	0.25	0.32	1.52	1.25	1.60	0.11	1.63	0.2	2.27	1.87	4.60
	0.1	0.04	0.43	1.25	2.67	0.01	2.82	0.2	5.79	2	10.7
